# A Genome Resequencing-Based Genetic Map Reveals the Recombination Landscape of an Outbred Parasitic Nematode in the Presence of Polyploidy and Polyandry

**DOI:** 10.1093/gbe/evx269

**Published:** 2017-12-18

**Authors:** Stephen R Doyle, Roz Laing, David J Bartley, Collette Britton, Umer Chaudhry, John S Gilleard, Nancy Holroyd, Barbara K Mable, Kirsty Maitland, Alison A Morrison, Andy Tait, Alan Tracey, Matthew Berriman, Eileen Devaney, James A Cotton, Neil D Sargison

**Affiliations:** 1 Wellcome Trust Sanger Institute, Hinxton, Cambridgeshire, United Kingdom; 2 Institute of Biodiversity Animal Health and Comparative Medicine, College of Medical, Veterinary and Life Sciences, University of Glasgow, United Kingdom; 3 Moredun Research Institute, Pentlands Science Park, Penicuik, United Kingdom; 4 Royal (Dick) School of Veterinary Studies, University of Edinburgh, United Kingdom; 5 Department of Comparative Biology and Experimental Medicine, Faculty of Veterinary Medicine, University of Calgary, Alberta, Canada

**Keywords:** F_1_ genetic map, *Haemonchus contortus*, ploidy, polyandry, pseudotestcross, recombination landscape

## Abstract

The parasitic nematode *Haemonchus contortus* is an economically and clinically important pathogen of small ruminants, and a model system for understanding the mechanisms and evolution of traits such as anthelmintic resistance. Anthelmintic resistance is widespread and is a major threat to the sustainability of livestock agriculture globally; however, little is known about the genome architecture and parameters such as recombination that will ultimately influence the rate at which resistance may evolve and spread. Here, we performed a genetic cross between two divergent strains of *H. contortus*, and subsequently used whole-genome resequencing of a female worm and her brood to identify the distribution of genome-wide variation that characterizes these strains. Using a novel bioinformatic approach to identify variants that segregate as expected in a pseudotestcross, we characterized linkage groups and estimated genetic distances between markers to generate a chromosome-scale F_1_ genetic map. We exploited this map to reveal the recombination landscape, the first for any helminth species, demonstrating extensive variation in recombination rate within and between chromosomes. Analyses of these data also revealed the extent of polyandry, whereby at least eight males were found to have contributed to the genetic variation of the progeny analyzed. Triploid offspring were also identified, which we hypothesize are the result of nondisjunction during female meiosis or polyspermy. These results expand our knowledge of the genetics of parasitic helminths and the unusual life-history of *H. contortus*, and enhance ongoing efforts to understand the genetic basis of resistance to the drugs used to control these worms and for related species that infect livestock and humans throughout the world. This study also demonstrates the feasibility of using whole-genome resequencing data to directly construct a genetic map in a single generation cross from a noninbred nonmodel organism with a complex lifecycle.

## Introduction

Recombination is a key genetic process: the breaking and rejoining of genetic material to produce novel genotypes and in turn, generate phenotypic variation. In eukaryotes, this is achieved by crossing-over between homologous chromosomes during the generation of gametes in meiosis. A common approach to studying recombination is to perform controlled matings (i.e., genetic crosses) between genetically distinct and inbred parents. The parents and offspring are then genotyped to construct genetic linkage maps, which aim to order genes or genetic markers based on the recombination frequency between them. This approach can also be used to identify regions of the genome underlying phenotypic variation, and has been widely used for mapping both simple and complex traits in a range of different organisms (Valentim et al. 2013; Zamanian et al. 2017). More recently, as whole-genome sequencing data have become available for many organisms, genetic maps have been used to inform or validate contig order in genome assemblies (Srinivasan et al. 2002, 2003; Opperman et al. 2008; Nemetschke et al. 2010; Thomas et al. 2012). Where a contiguous genome assembly is already available, a linkage map can be used to explore variation in recombination rates throughout the genome (Rockman and Kruglyak 2009) and determine how this has shaped other aspects of genome architecture, such as the distribution of repeats or the impact of natural selection.

Understanding variation in the rate and pattern of recombination is crucially important in the design and analysis of experiments aimed at mapping the genetic basis of phenotypic traits and in interpreting genetic variation in natural populations. Between species, a negative relationship between genome size and recombination rate has been described (Lynch 2006). Within a species, variation in recombination rate is strongly influenced by the sex of the organism; recombination may not occur in one of the two sexes (typically the heterogametic sex, i.e., the Haldane–Huxley rule; Burt et al. 1991), or, if recombination does occur in both sexes, then females tend to exhibit higher recombination rates than males (i.e., heterochiasmy; Lenormand and Dutheil 2005). In addition, recombination rates have been shown to vary considerably within and between chromosomes, which has been attributed to genomic features including but not limited to GC content, gene density, gene size, simple repeats, and chromatin state (Barnes et al. 1995; Beye et al. 2006; Chan et al. 2012; Tortereau et al. 2012). Among nematodes, recombination is best characterized in the model organism *Caenorhabditis elegans*, where direct comparison of the physical and genetic maps clearly reveals asymmetrically distributed high and low recombination rate domains in each chromosome, correlated with low and high gene density (and gene expression), respectively (Barnes et al. 1995; Rockman and Kruglyak 2009; Kaur and Rockman 2014). However, even in this model organism, the precise local DNA features that mediate these rate changes remain unclear. Less is known about recombination in parasitic helminths. Low density genetic maps are available for only three species, the root knot nematode *Meloidogyne hapla* (Opperman et al. 2008; Thomas et al. 2012), the human blood-fluke *Schistosoma mansoni* (Criscione et al. 2009), and the rat gastrointestinal parasite *Strongyloides ratti* (Nemetschke et al. 2010), and only discrete regions of recombination variation have been described in *M. hapla* (Thomas et al. 2012). Recombination rate variation has been proposed to influence the distribution of genetic variation, and in turn, evolution of phenotypic traits in *C. elegans* (Cutter and Payseur 2003; Rockman et al. 2010; Andersen et al. 2012). Therefore, understanding genome-wide recombination variation in parasitic species will likely be important in predicting the genetic architecture and evolution of important parasite life history traits, including pathogenicity, response to host immunity, and chemotherapeutic selection.

The parasite *Haemonchus contortus* is among the most pathogenic of the gastrointestinal nematodes and exerts significant burdens on animal health and the economic viability of livestock farming (Urquhart 1996). It is also an emerging model for the biology of parasitic helminths more widely, particularly for understanding anthelmintic drug action and resistance (Gilleard 2013). *H. contortus* makes a particularly good model because 1) it is the most genetically tractable of any of the strongylid (clade V) parasitic nematodes, a large and important group of parasites including key human and veterinary pathogens; 2) it is a sexually reproducing diploid organism for which the karyotype—five autosomes and XX/XO sex chromosomes—is well defined (Bremner 1954; Redman et al. 2008a); 3) two published draft genome sequences and extensive transcriptomic data are available (Laing et al. 2013, 2016; Schwarz et al. 2013); 4) it is highly fecund, making propagation of crosses easier; 5) it is amenable to cryopreservation of isolates; and 6) it is one of the few parasitic nematode species in which genetic crosses have been successfully established (Le Jambre 1977; Le Jambre et al. 1979, 2000, 2005; Sangster et al. 1998; Hunt et al. 2010; Redman et al. 2012).

Anthelmintic drug failure is an important economic and animal health problem, as anthelmintic resistance is widespread on many farms, and populations and isolates resistant to most major broad-spectrum classes of anthelmintics have been described (Kotze et al. 2014; Mederos et al. 2014; Sales and Love 2016). Accordingly, significant research effort is focused on the development of novel anthelmintics (Kaminsky et al. 2008) or vaccines (Bassetto and Amarante 2015) for parasite control. Although research on *H. contortus* has been instrumental in understanding some of the mechanisms by which resistance arises (Kotze et al. 2014; Britton et al. 2016), the genetic basis of resistance remains largely unresolved and is likely complex. For example, while resistance to benzimidazoles—the class of anthelmintics for which the genetic basis of resistance is best understood—has been linked clearly to mutations at three sites in the isotype-1 β-tubulin gene (Kwa et al. 1994; Silvestre and Cabaret 2002; Ghisi et al. 2007), there is evidence that it is a more complex trait than previously assumed (Zamanian et al. 2017). In contrast, genome-wide studies of ivermectin response—another major anthelmintic—in a number of parasitic helminth species support the hypothesis that this is a quantitative, multigenic trait (Bourguinat et al. 2015; Choi et al. 2017; Doyle et al. 2017). Therefore, establishing the genomic context in which drug resistance alleles are inherited using *H. contortus* will help to resolve the mechanisms by which resistance evolves and spreads in other species of parasitic nematodes as well.

The purpose of this study was to produce a genetic map of *H. contortus*, initially in order to establish an anchored framework for a draft genome under development, and subsequently to estimate the frequency and distribution of recombination in the genome. To do so, we performed a cross between two genetically divergent strains of *H. contortus* that differed in their anthelmintic resistance phenotypes: one that was fully susceptible (Redman et al. 2008b) and one that showed high levels of resistance to three commonly used anthelmintics (Williamson et al. 2011). Four constraints restrict use of *H. contortus* crosses to implement standard classical approaches for genetic mapping: 1) there is an extremely high level of sequence polymorphism present both in field and laboratory strains of *H. contortus* (Gilleard and Redman 2016); 2) few very highly inbred isolates are available to use as parents, and so isolates comprise multiple genotypes; 3) it is difficult, although not impossible, to perform single parent crosses from inbred lines (Sargison et al. 2017); and 4) mating is polyandrous, that is, multiple males can, and will, mate with a single female (Redman et al. 2008a). To overcome these limitations, we developed a genomic strategy to characterize segregating single nucleotide polymorphisms within families based on variants present in a single female and her progeny, which was subsequently used to construct an F_1_ genetic map and explore the recombination landscape of the genome. We discuss the implications of recombination, and other novel life history traits identified here, in the context of generating and maintaining genetic variation in parasite populations, and how these factors might impact the spread of anthelmintic resistance in this species.

## Materials and Methods

### Ethics Approval and Consent to Participate

All experimental procedures described in this manuscript were examined and approved by the Moredun Research Institute Experiments and Ethics Committee and were conducted under approved UK Home Office licenses in accordance with the Animals (Scientific Procedures) Act of 1986. The Home Office licence number is PPL 60/03899 and experimental code identifier was E46/11.

### Construction of the Genetic Cross and Collection of Worm Samples

**Fig. 1.**
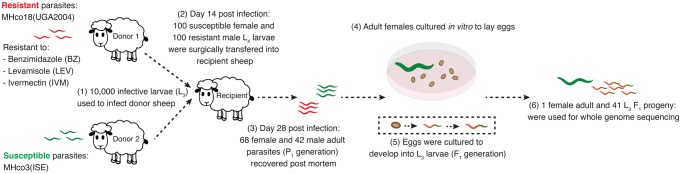
—Outline of genetic cross between MHco3(ISE) drug susceptible and MHco18(UGA2004) multidrug resistant *Haemonchus contortus*. A total of 68 MHco3(ISE) females and 42 MHco18(UGA2004) males (from an infection of 100 individuals of each sex) were recovered post mortem (P_1_ generation), after which reproductively mature females were incubated in vitro to lay eggs that were subsequently cultured to L_3_ stage. These larvae represent the F_1_ generation of the cross.

A schematic of the experimental genetic cross is outlined in figure 1. Briefly, two parasite naïve lambs were each infected with ∼10,000 infective larvae from one of two ovine-derived *H. contortus* strains, the anthelmintic susceptible MHco3(ISE) (Redman et al. 2008b), or MHco18(UGA2004) (Williamson et al. 2011), a multidrug resistant strain that is insensitive to standard manufacturers recommended dose rates of benzimidazole, imidazothiazole, and macrocyclic lactone anthelmintics. At 14 days postinfection (DPI), developing sexually immature parasitic stages were recovered post mortem, and the sex of the L_4_ stage immature adults was determined by microscopic examination of gross morphology (Denham 1969; Ministry of Agriculture Fisheries and Food 1971). A total of 100 MHco3(ISE) female and 100 MHco18(UGA2004) male L_4_ (P_1_ generation) were surgically transferred into the abomasum of a recipient sheep to allow reproduction that would generate F_1_ hybrid progeny between the two strains. At 28 DPI, 67 MHco3(ISE) females and 42 male MHco18(UGA2004) P_1_ from the recipient sheep were recovered post mortem, after which the males were snap frozen in liquid nitrogen and stored. Sampling was performed at 28 DPI to ensure that all of the females would have mated, and that they would be mature enough to have more viable progeny than is thought to be the case in early patency. Individual females were placed into individual wells of 24-well cluster plates (Sarstedt) containing 1 ml of warm RPMI 1640 cell culture media containing 1% (v/v) d-glucose, 2 mM glutamine, 100 IU/ml penicillin, 100 mg/ml streptomycin, 125 mg/ml gentamycin, 25 mg/ml amphotericin B (Redmond et al. 2006), and Hepes (1% v/v) and incubated in 5% CO_2_ at 37 °C for 48 h to promote egg shedding. Eggs were transferred at 24 and 48 h and mixed with fresh helminth egg-free sheep faeces before being incubated at 24 °C for 2 weeks to allow larval development to L_3_. After this time, a single female parent (P_1_) and a total of 41 F_1_ L_3_ progeny were individually stored in preparation for DNA extraction and sequencing library preparation.

### Sample Preparation and Sequencing

The female parent was dissected on ice to isolate the head from the body (in three sections, as three technical replicates) to avoid contamination with fertilized eggs present *in utero*. The female sections and individual L_3_ were transferred into 10 µl of sample lysis buffer (working solution: 1000 µl Direct PCR Lysis Reagent [Viagen, Los Angeles], 50 µl 1 M DTT, 10 µl 100 mg/ml Proteinase K) in a 96-well plate and allowed to incubate at 60 °C for 2 h followed by 85 °C for 45 min. Whole genome amplification (WGA) of each sample lysate was performed using RepliG amplification. First, 2–5 µl of sample lysate was combined with 5 µl of 1.3 M Trehalose in a 96-well plate and mixed by gentle tapping, incubated for 3 min at 95 °C, and placed on ice. A 40-µl REPLI-g amplification mix (29 µl REPLI-g Reaction Buffer + 1 µl REPLI-g polymerase + 10 µl 1.3 M Trehelose) was added to each well, and incubated for 16 h at 30 °C followed by 10 min at 65 °C before being placed on ice. The WGA DNA was cleaned using Ampure XP beads at a 1.4× bead: DNA reaction ratio, before being eluted in 50 µl of RNase/DNase-free water and stored at 4 °C.

PCR-free sequencing libraries (mean length of ∼400 bp) were prepared by methods previously described (Kozarewa et al. 2009) and sequenced on an Illumina HiSeq X10, resulting in ∼3.06 × 10^9^ 151-bp paired-end reads (see supplementary table S1, Supplementary Material online, for a breakdown of reads per lane and per sample). Raw sequence data are archived under the ENA study accession ERP024253.

### Mapping and Variant Analysis

Raw sequence data were mapped to the current unpublished version of the reference genome for *Haemonchus contortus* (v3.0, available at ftp://ngs.sanger.ac.uk/production/pathogens/Haemonchus_contortus) using *Smalt* (http://www.sanger.ac.uk/science/tools/smalt-0) with the mapping parameters “-y 0.8 -i 800.” Data from multiple sequencing lanes for a single sample were merged (*samtools-1.3 merge*) and duplicate reads removed (*Picard v2.5.0*; https://github.com/broadinstitute/picard) from the bam files before further processing.

Variants were called using *GATK Unified Genotyper* (v3.3.0) (McKenna et al. 2010). The raw variant set was initially filtered to flag variants as low quality if they met the following conditions: quality by depth (QD) < 2; Fisher’s test of strand bias (FS) > 60; RMS mapping quality (MQ) < 40; rank sum of alt versus reference mapping quality (MQRankSum) < −12.5; read position rank sum (ReadPosRankSum) < 8; read depth (DP) < 10. Variants were filtered further using *vcftools* (v0.1.14) (Danecek et al. 2011) to exclude sites with low quality flags, minimize loci with missing data (“—max-missing 0.8”), exclude indels (“—remove-indels”), exclude SNPs with genotype quality (GQ) < 30, and ensure sites were biallelic (“—min-alleles 2, —max-alleles 2”). A gff file generated from *RepeatMasker* of the reference genome was also used to filter variants from the vcf file that were likely associated with repetitive and difficult to map regions of the genome.

Sex determination of the F_1_ progeny was performed by measuring: 1) the relative ratio of autosome to X chromosome (characterized and thus named based on synteny with *C. elegans* autosomes and X chromosome) read depth calculated using *samtools-1.3 bedcov*; and 2) the relative heterozygosity of the X chromosome using *vcftools* (v0.1.14) “–het.”

### Genetic Map Construction

A “pseudotestcross” (PT) strategy (Grattapaglia and Sederoff 1994) was employed to generate the genetic map, which required that each input variant site was: 1) heterozygous in the female parent, and 2) segregating in a 1:1 genotype ratio in the F_1_ progeny. The segregation pattern of each SNP was first calculated in the F_1_ progeny (with “A” referring to the reference allele and “a” to the nonreference variant allele), which resulted in SNPs being placed into one of four categories that best described the likely genotypes of the parents of the cross for that given SNP: 1) “PT: 110,” that is, AA × Aa, 2) “PT: 011,” that is, Aa × aa, 3) “intercross,” that is, Aa × Aa, or 4) SNPs that were clearly segregating in the brood, but for which the segregation ratio of genotypes in the progeny did not fit a simple Mendelian segregation pattern that could be generated via reproduction from a single pair of parents. SNP density was further reduced using *vcftools* (v0.1.14) (Danecek et al. 2011)*—thin* as described in the text. The number of filtered SNPs per segregation group is described in supplementary table S2, Supplementary Material online. Genotypes for autosomal PT: 011 and PT: 110 SNPs that were heterozygous in the female parent were imported into *R-3.2.2* (R Core Team 2015), after which pairwise recombination fractions (RF) and logarithm of the odds (LOD) scores were determined for each chromosome using *R/QTL* (Broman et al. 2003). Recombination fractions were converted into map distance in centimorgans (cM) using the kosambi map function. Variants resulting in inflation of map distances were identified using *qtl::droponemarker*, and as outliers relative to surrounding markers via visual inspection of LOD and RF using *qtlcharts*::*iPlot* (Broman 2015). These aberrant markers were removed in the generation of the final map.

A reverse cross design, whereby SNPs were chosen that: 1) segregated in a 1:1 genotype ratio and 2) were homozygous in the female parent (and therefore putatively heterozygous in the male parents) was also performed. Although polyandry prevented a male-specific genetic map from being constructed (multiple male parents confounded the calculation of linkage between heterozygous sites), these data were used to determine the segregation frequency of alleles from the male parents.

### Recombination Landscape

Recombination patterns for each chromosome were visualized first by generating genotype matrices of pseudotestcross markers for each chromosome using *vcftools* (v0.1.14) “—012,” followed by visualization using the *gplots:*: *heatmap2* function in R. These maps highlighted recombination breakpoints, linkage blocks, and regions of excess heterozygosity or reduced heterozygosity. Recombination rate changes throughout the genome were visualized by constructing Marey maps, which compare the position of the marker in the genome (base position in the fasta sequence) to the relative position in the genetic map. A fitted loess smoothed line of the genetic map positions in 1-Mb windows was performed to calculate the recombination rate.

### Kinship Analysis

Analysis of genetic relatedness between F_1_ progeny was undertaken to characterize evidence of polyandry and to determine, if present, the impact on the cross analysis. Principal component analysis (PCA) of genetic distances between the F_1_ progeny and female parent was performed using the *SNPrelate* package in R 3.1.2 (Zheng et al. 2012). Kinship coefficients were determined for all pairwise relationships among the F_1_ progeny using *KING* (Manichaikul et al. 2010). Relationship networks of the pairwise kinship coefficients were visualized using *Gephi* (v 0.9.1; Bastian et al. 2009) to highlight full- and half-sib relationships among the F_1_ progeny. Layout of the kinship network graph was determined using the *Force Atlas* parameter, with the nodes (F_1_ individuals) colored by their proposed kinship group, and the thickness of the edges proportionate to the kinship coefficient between two F_1_ individuals (nodes).

## Results

### Genome Sequencing and Genetic Diversity of a Genetic Cross between Two Isolates of *H. contortus*

A genetic cross was performed between two genetically and phenotypically defined *H. contortus* strains: females were from MHco3(ISE), a serially passaged anthelmintic susceptible “laboratory” strain that has been well characterized by genomic and transcriptomic analyses (Laing et al. 2013, 2016), and males were from MHco18(UGA2004), a multidrug resistant serially passaged strain originally isolated from the field at the University of Georgia (Williamson et al. 2011) (fig. 1). Whole genome sequencing (WGS) was performed on DNA derived from a single adult MHco3(ISE) female parent and 41 of her F_1_ L_3_ progeny to achieve a minimum 30× sequencing coverage per sample (mean sequencing depth: 34.80× ± 16.16 SDs), generating a median yield of 65.97 million reads per sample (supplementary table S1, Supplementary Material online). Mapping of the sequencing data was performed using an improved genome assembly of the MHco3(ISE) isolate described by Laing et al. (2013), which now consists of five scaffolds representing the autosomal chromosomes and two scaffolds representing the X chromosome, for an assembly length of ∼279 Mb. Sequence depth of the X chromosome scaffolds relative to the five autosomal scaffolds, together with rates of heterozygosity on the X chromosome scaffolds, revealed 20 male and 21 female F_1_ progeny in the brood.

**Fig. 2.**
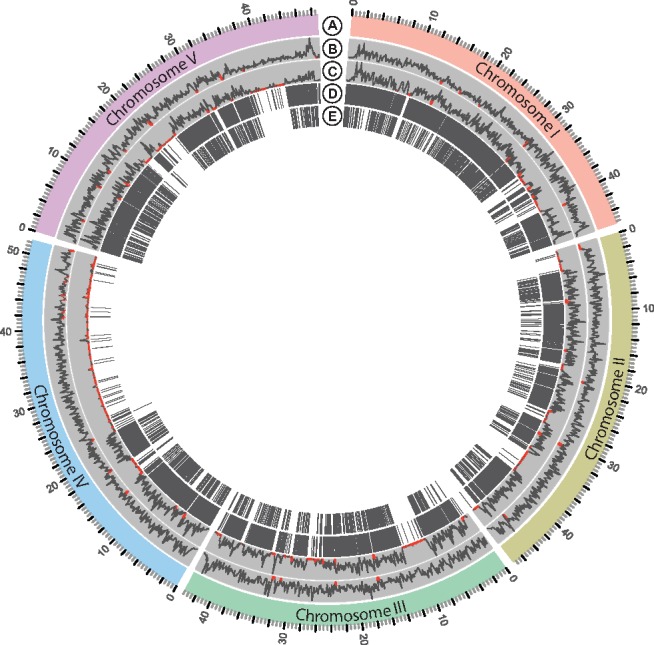
—Autosome-wide variant density and candidate genetic map markers identified from the female parent and F_1_ progeny. (*A*) The five autosomes of *Haemonchus contortus*, named based on synteny with *Caenorhabditis elegans* chromosomes, span 237 Mb. (*B*) SNP density was calculated in 100-kb windows, and is presented as the relative variant density of the female parent (P_1_) and all F_1_ progeny. (*C*) Density of heterozygous variants in the female parent. (*D*) Positions of candidate pseudotestcross SNPs that were heterozygous in the female parent and segregated in a 1:1 genotype ratio in the F_1_ progeny. Red annotations in plots (*C*) and (*D*) highlight low density regions, defined as genome-wide mean SNP density minus 3 SD. (*E*) Positions of the final set of 1,618 SNPs used in the F_1_ genetic map. The plot was produced using *Circos* (Krzywinski et al. 2009).

Approximately 5.3 million single nucleotide polymorphisms (SNPs) that passed stringent filtering criteria were identified in the autosomal chromosomes (fig. 2*A* andsupplementary table S2, Supplementary Material online), at a genome-wide density of 2,242 SNPs per 100 kb (fig. 2*B*), or 1 SNP per 44.6 base pairs (bp). A pseudotestcross approach was used to generate the F_1_ genetic map, which required that candidate markers: 1) were heterozygous in the female parent; and 2) segregated in a ratio statistically indistinguishable from a 1:1 genotype ratio in the F_1_ progeny. By using these criteria, we identified a set of markers that could be analyzed using the same statistical approaches as conventional linkage mapping using a test cross. Analysis of the 730,825 heterozygous SNPs in the female MHco3(ISE) parent demonstrated that the distribution of variation was not uniform throughout the genome, with a number of long contiguous regions of homozygosity observed (fig. 2*C* and supplementary fig. S1, Supplementary Material online). In particular, ∼27 Mb of the second half of chromosome IV was largely homozygous, containing ∼50% more homozygous variant sites and ∼30% less heterozygous sites compared with the genome-wide average (supplementary table S3, Supplementary Material online).

Among the SNPs that were heterozygous in the female parent, 171,876 SNPs segregated at an ∼1:1 genotype ratio in the F_1_ progeny (supplementary table S2, Supplementary Material online; PT: 110 and PT: 011). To avoid including tightly linked SNPs, the 171,876 candidate SNPs were thinned to 1 per 25,000 bp, which resulted in a final candidate list of 5,595 SNPs for analysis in the cross.

### Characterization of an Autosomal F_1_ Genetic Map Generated Using Pseudotestcross SNP Markers

Initial analysis of genome-averaged genotype ratios (supplementary fig. S2, Supplementary Material online) of the candidate pseudotestcross sites in each F_1_ individual revealed that most individuals displayed an approximate 50:50 ratio of homozygous: heterozygous genotypes, as expected. However, seven individuals presented as outliers with an excess of heterozygous genotypes (supplementary fig. S2*A*, Supplementary Material online; moderate outliers: individuals F1_12, F1_30, F1_40; extreme outliers: individuals F1_21, F1_23, F1_32, F1_38). The variant-allele frequency distribution of these individuals (supplementary fig. S3, Supplementary Material online) revealed a skew consistent with a nondiploid complement of chromosomes, with a major nonreference (relative to the genome assembly) allele frequency peak at ∼30% and minor peak at 60% frequency. This allele frequency skew was typically found across all chromosomes within an individual, suggesting that they were not aneuploids. A notable exception was individual F1_30 (one of the moderate outliers), where chromosomes I, III, and V had a distinct allele frequency spectrum consistent with more than two copies of each chromosome present, relative to chromosomes II and IV, which appeared to be disomic. All seven of these nondiploid individuals were therefore removed from the pseudotestcross analysis (supplementary fig. S2*B* and *D*, Supplementary Material online; *n* = 34).

**Fig. 3.**
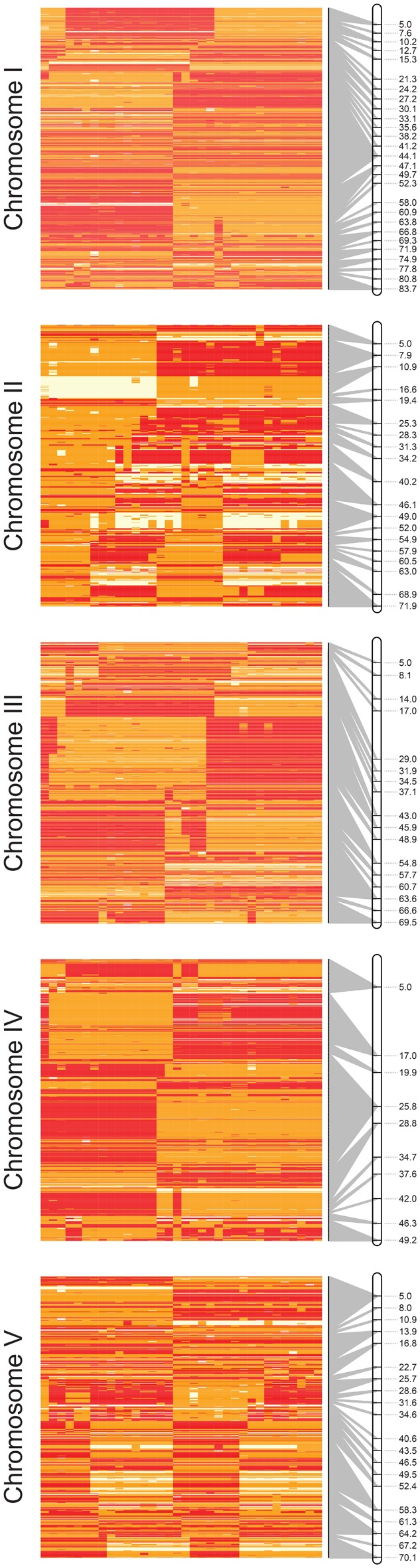
—Recombination and genetic maps of the five autosomes of *Haemonchus contortus*. Recombination plots depict genotype segregation patterns per F_1_ progeny (columns; clustered by genetic similarity) of pseudotestcross markers used in the genetic map (rows). Segregating “parental” and “recombinant” haplotypes inherited from the female parent are indicated by opposing color schemes. Genotypes: AA: red; Aa: yellow; aa: white. The relationship between SNP position in the recombination map and genetic map position (cM) is represented by a connecting gray line; multiple SNPs between which no recombination was observed collapse into a single map position in the genetic map (gray ribbon from multiple SNPs to a single map marker).

A reanalysis of the remaining 34 individuals revealed 217,575 pseudotestcross SNPs, 129,985 intercross SNPs, and 383,265 SNPS that were heterozygous in the female parent but did not segregate in a way compatible with analysis as a single-pair mating cross (supplementary table S2, Supplementary Material online). Thus, a total of 4,587 pseudotestcross SNPs (217,575 SNPs thinned to 1 SNP per 25,000 bp) were candidate markers for the map construction using R/QTL (fig. 2*D*), from which 1,618 SNPs were used in the final genetic map (fig. 2*E* and supplementary table S4, Supplementary Material online). Recombination plots and genetic maps for the five autosomes are presented in figure 3, and characteristics of the map are presented in table 1. The total map distance of the five autosomes was ∼344.46 cM. The number of markers per chromosome ranged from 215 on chromosome II to 475 on chromosome I, with a mean value of 323.6 markers per chromosome. Significant gaps in the map correlated with absence, or very low density, of the prerequisite heterozygous SNPs in the female parent, as described earlier (fig. 2*C*). This loss of markers was most obvious in chromosome IV, where only approximately half of the chromosome is represented in the map, resulting in a map length of 49.21 cM, compared with the average map length of other chromosomes of 73.79 cM. The genome-wide recombination rate was on an average 604.12 (± 84.01 SD) kb/cM or 1.68 (± 0.25 SD) cM/Mb, which corresponded to an overall average number of crossover events per chromosome of 0.69 (± 0.12 SD). Chromosome IV was again an outlier, with a recombination rate of 2.01 cM/Mb, ∼21% higher than the other four autosomes (1.68 cM/Mb average). Analysis of the X chromosome diversity from the adult female and all progeny revealed 100,016 SNPs in the 23.3- and 18.9-Mb X-linked scaffolds; this frequency (1 SNP per 422 bp) equates to ∼10-fold fewer variable sites on the X chromosome relative to the autosomes. Attempts to generate an X chromosome genetic map were limited by a lack of prerequisite heterozygous variant sites in the female X chromosome sequences (supplementary fig. S1, Supplementary Material online). To explore this further, the diversity of hemizygous genotypes called in the male F_1_ progeny, that is, genotyped as AA or aa reflecting the haploid X^A^ or X^a^ allele, respectively, was compared with genotypes resolved in the female parent (supplementary fig. S4, Supplementary Material online). Strikingly, male genotypes were entirely concordant with the female parent, further supporting the lack of segregating genetic diversity in the female parent diploid X chromosomes. Female F_1_ progeny contained both homozygous and heterozygous sites in their X chromosomes; given the lack of variation in the female parent, this diversity was entirely inherited from the paternal X chromosome.

**Table 1:** Summary Characteristics of the F_1_ Genetic Map, Including Number of Markers Used, Map Length, Recombination Rate, and Crossover Frequency

Chromosome	Chromosome Length (bp)	Markers Used (#)	Genetic Map Length (cM)	Recombination Rate (kb/cM)^a^	Recombination Rate (cM/Mb)^b^	Crossovers per Chromosome^c^
I	45778363	475	83.71	546.87	1.83	0.84
II	47384193	215	71.88	660.13	1.51	0.72
III	43564237	363	69.53	626.55	1.60	0.70
IV^d^	51819793	226	49.21	490.85	2.04	0.49^e^
V	48825595	339	70.13	696.22	1.44	0.70
Total/average	237372181	1618	344.46	604.12	1.68	0.69

a Recombination rate (kb/cM): chromosome length (kb)/genetic map length.

b Recombination rate (cM/Mb): genetic map length/(chromosomal length/10^6^).

c Crossovers per chromosome: (genetic map length/100)/number of chromosomes.

d The genetic map only spanned ∼24 Mb of chromosome IV due to homozygosity in the female parent. As such, recombination rates have been calculated for chromosome IV using 24154752 bp (position of the genetic map marker closest to the homozygosity region) as the chromosome length.

e Likely to be underestimated given only half of the chromosome is present.

### Patterns of Recombination within Autosomal Chromosomes of the F_1_ Progeny

**Fig. 4.**
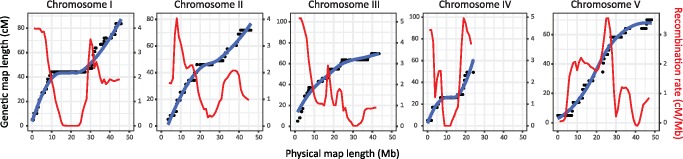
—Analysis of recombination rate variation throughout the genome. Marey maps were constructed to show the relationship between the genetic position of each marker (black point) relative to the physical position of the marker in the genome. Line of best fit was plotted using default parameters of the *geom_smooth* function of *ggplot2* in R. Recombination rates (cM/Mb; red line) were calculated by calculating genetic map distance in 1-Mb windows throughout the genome from a fitted *loess*-smoothed line of the genetic map positions.

Analysis of recombination rate throughout each chromosome was determined by comparing physical and genetic distances, which can be visualized in a Marey map (Chakravarti 1991) (fig. 4). Recombination rate (fig. 4 red line; cM/Mb) was not uniform throughout the chromosomes, nor was it consistent between chromosomes. Chromosomes I, II, and IV tended to show a pattern of three main recombination rate domains; a reduced recombination rate domain toward the middle of the chromosome, flanked by domains of increased recombination rate that extend toward the ends of the chromosomes. This three-domain pattern was not as clear for chromosomes III and V; chromosome III showed a greater recombination rate in the first half of the chromosome that decreased throughout the second half of the chromosome, whereas chromosome V had longer low recombination rate domains toward the ends of the chromosome arms, and greater recombination rate toward the middle of the chromosome. It is curious that chromosome IV retained the three-domain recombination architecture, given that the right arm is largely missing due to lack of the prerequisite heterozygous sites in this region of the female parent (fig. 2*C* andsupplementary fig. S1, Supplementary Material online). Each chromosome also showed evidence of additional low recombination rate domains at one or both ends of the chromosome in the subtelomeric regions extending into the chromosome. Finally, within the elevated recombination rate domains, the recombination rate was not necessarily constant; discrete peaks of high recombination rates were observed in all chromosomes. However, the relative positions of these high recombination peaks were not the same between chromosomes.

### Family Structure and Kinship among the Brood

**Fig. 5.**
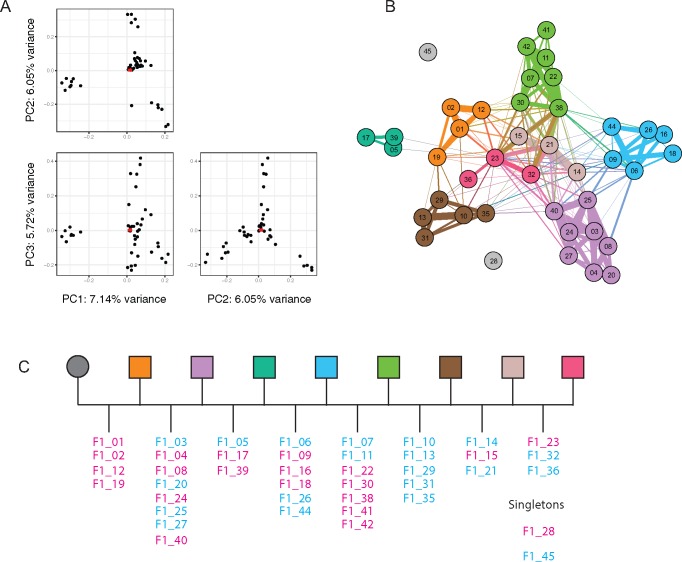
—Familial relationships determined via analysis of genetic diversity and kinship between full- and half-sibs. (*A*) Principal component analysis of parent and progeny genetic diversity, comparing the top three principal components (PCA). The female parental values (*n* = 3) are indicated as red points in each plot. (*B*) Network analysis of kinship coefficients determined by *KING* (Manichaikul et al. 2010) and visualized by *Gephi* (Bastian et al. 2009) highlighting full-sib relationships between progeny. The thickness of the line (edges) represents the kinship coefficient between individuals (nodes) and is proportionate to the relationship between pairs. (*C*) Proposed pedigree of the brood. Full-sib male (blue) and female (pink) progeny are indicated for each subfamily. Colors used in (*B*) and (*C*) represent groups of progeny that share a common father.

*Haemonchus contortus* is known to be polyandrous (Redman et al. 2008a). This knowledge, together with the observation that >50% of SNPs did not segregate in either a 1:1 or 1:2:1 genotype ratio (supplementary table S2, Supplementary Material online), suggested that the 41 progeny analyzed were sired from more than a single male parent. An initial analysis of genetic relatedness by principal component analysis (PCA) of 21,822 autosomal SNPs (complete data set thinned using a linkage disequilibrium threshold of 0.5 and minor allele frequency of 0.05) revealed obvious genetic structure, with at least four (PC 1 vs. 2) to as many as six (PC 2 vs. 3) putative clusters of F_1_ progeny (fig. 5*A*), consistent with the hypothesis that the brood resulted from polyandrous mating.

To more accurately describe these putative relationships among the progeny, we calculated kinship coefficients (Manichaikul et al. 2010), which describe the probability that a given allele in two individuals is identical by descent (i.e., an allele shared due to recent shared ancestry, as opposed to identical by state, in which the allele is simply shared by two individuals without common ancestry), for all pairwise combinations of progeny. Employing all autosomal SNPs (*n* = 5,323,039 SNPs), this analysis revealed eight clusters of full-sib relationships containing multiple F_1_ progeny (fig. 5*B*). Two individuals, F1_28 and F1_45, did not share any pairwise kinship coefficients consistent with a full-sib relationship with any individual, and hence, may represent the progeny from additional paternal contributions to the brood. Three individuals, F1_21, F1_23, and F1_38, seemed to show full-sib relationships with individuals from multiple families via strong kinship associations between themselves and others. Intriguingly, these were the same individuals identified as outliers with excess heterozygosity (supplementary fig. S2, Supplementary Material online) and that showed a skewed allele frequency distribution (supplementary fig. S3, Supplementary Material online) suggestive of aneuploidy or polyploidy. These autosomal kinship data are further supported by the observation that X chromosome diversity in the female F_1_ progeny, which reflects paternal X chromosome inheritance in the absence of maternal X chromosome diversity, clusters the female F_1_ progeny into five groups of two or more individuals (supplementary fig. S4, Supplementary Material online). Three unclustered individuals were also identified for the X chromosomes, including individual F1_28, which did not share any full-sib relationships in the kinship analysis (fig. 5*B*). These X chromosome derived clusters are concordant with the full-sib family structure using autosomal SNPs. Taken together, these data describing the familial relationships among the F_1_ progeny cohort lead us to propose a pedigree consisting of at least eight paternal contributions (fig. 5*C*).

## Discussion

Our comprehensive genetic characterization of genome-wide patterns of segregation in progeny from a brood of parasites revealed extensive variation in recombination rates across chromosomes, and confirmed previous suggestions of polyandry as the dominant mating system in *H. contortus* (Redman et al. 2008a). Moreover, analysis of genetic variation in both autosomes and the X chromosome identified extended regions of reduced heterozygosity in the female parent, which could be a genetic consequence of population bottlenecks during the generation and maintenance of the MHco3(ISE) line. Analysis of allele frequency spectra also suggested the presence of polyploids among the progeny. The availability of a largely complete chromosomal scale *H. contortus* genome assembly facilitated such analyses. Here, we discuss some of the characteristics and challenges associated with the assembly of a genetic map when homozygous single parent crosses are not available, and how some of the features of the genetic cross impact on our understanding of *H. contortus* biology and anthelmintic resistance.

### Prediction of Genomic Structure

A small number of genetic linkage maps have been described for free-living nematodes (*Caenorhabditis elegans* and *Pristionchus pacificus*), parasitic nematodes (*Meloidogyne hapla, Strongyloides ratti*), or parasitic trematodes (*Schistosoma mansoni*). *Haemonchus contortus* was found to have the lowest genome-wide recombination rate among these worms, at an average of 1.68 cM/Mb throughout the ∼279-Mb genome. However, the recombination rate tended to scale proportionately with genome size, that is, larger genomes have lower recombination rates (supplementary fig. S5, Supplementary Material online). Although the recombination rates of these worms are somewhat lower than predicted by a model describing the relationship between eukaryotic genome size and recombination rate (supplementary fig. S5, Supplementary Material online, gray dashed line) (Lynch 2006), they are more consistent with recombination rates of other invertebrates (supplementary fig. S5, Supplementary Material online, gray points; see Supplementary Table 1 from Lynch 2006 for invertebrate recombination rate data). This relationship between genome size and recombination rate is somewhat dependent on the number of crossovers per chromosome per meiosis; for example, in *C. elegans*, almost complete crossover interference occurs, such that only a single crossover per pair of homologous chromosome is observed (Meneely et al. 2002). In *H. contortus*, some but certainly not complete interference was observed, with an average rate of 0.69 crossovers per chromosome (i.e., 1.38 crossovers per pair of homologous chromosomes). Given that the genetic map of *P. pacificus* (a free-living clade V nematode often compared with *C. elegans* but more distantly related to *C. elegans* than *H. contortus*) is expanded relative to *C. elegans* due to the presence of double crossovers (Srinivasan et al. 2002; Hong and Sommer 2006), the observation of complete chromosome interference in *C. elegans* is likely to be an exception rather than the rule. The mechanisms by which recombination rates in free living or parasitic species are controlled is largely unknown. However, it is clear that there is significant diversity among the helminths: from single to few crossovers per homologous chromosomes per meiosis in *C. elegans* and *H. contortus/P. pacificus*, respectively, to multiple chiasmata between homologous pairs in *S. mansoni* (Hirai et al. 1996), and in an extreme case, recombination between all four chromatids within a homologous pair in *M. hapla* (Liu et al. 2007). The presence of such diversity provides an insight into the evolutionary potential of these species, and perhaps an opportunity to explore the mechanisms of recombination rate variation via comparative genomic approaches.

To our knowledge, we are the first to report the use of whole genome sequencing to construct a genetic map of any helminth species. WGS allowed significantly greater flexibility in choosing high quality variants to be included in the genetic map than other marker-based approaches such as amplified fragment length or Sanger-sequencing derived markers, and more recently, higher throughput RADseq and genotype-by-sequencing approaches, and allowed us to fully exploit the genetic variation in the available progeny. This was particularly important given that: 1) the progeny were not derived from a cross between genetically distinct homozygous single male and female parents, as is typical for a genetic mapping experiment; 2) the high genetic diversity within isolates meant that a lot of markers have to be screened and discarded to find “biallelic markers” that segregate appropriately for analysis; and 3) we did not know how many males would contribute to the progeny of the cross due to polyandry. As such, we developed a bioinformatics pipeline to select markers based on the genotype segregation ratio of the progeny (approximate 1:1 genotype ratios: Aa: aa [PT: 011] or AA: Aa [PT: 110]) and heterozygous sites in the female. This unusual cross design, chosen to account for the biological complexity, meant that relatively few of the sites that differed between parents were usable in the map (pseudotestcross SNPs represent only 4.09% of the total SNPs in the brood, and 29.77% of SNPs heterozygous in the female parent, before deliberate thinning). A very large panel of traditional markers would thus have been required even for the relatively small number of progeny analyzed here. The genome-wide resequencing approach that we used would seem to be the only practical way to generate complete recombination maps in this system. Genome-wide genetic variation that has been validated as segregating in a Mendelian fashion also provides a valuable resource for downstream experiments such as: QTL analyses of parasite traits (e.g., drug resistance); using individuals phenotyped in vitro using bioassays (Le Jambre 1976; Coles et al. 1988; Hunt and Taylor 1989; Álvarez-Sánchez et al. 2005); or as a source of genome-wide population genetic markers, which typically require low/no linkage disequilibrium between loci.

We initially intended to use the F_1_ genetic map to guide improvements of the assembly of the draft genome for *H. contortus* MHco3(ISE) (Laing et al. 2013); while subsequent improvements to the genome assembly have rendered this unnecessary, the colinearity of the genetic and physical maps confirms the accuracy of the current assembly. A number of features of this data set would not have been obvious without integrating the genetic map and physical assembly. The first of these includes the nonuniform distribution of genetic map markers in the genome. This is most obvious in chromosome IV in which approximately half of the chromosome is missing from the genetic map, due to a long tract of homozygosity in the female parent. However, each chromosome contained multiple megabase-scale gaps that directly corresponded to a deficiency of heterozygosity in the female parent in these regions. This may reflect the genetic history of this particular strain: MHco3(ISE) is a laboratory strain (Redman et al. 2008b) derived from ISE, a strain that was originally generated by 15 rounds of half-sib matings of an outbred isolate (Roos et al. 2004). Since that time, MHco3(ISE) has been passaged and cryopreserved on numerous occasions at an unknown, but likely limited, population size. Although significant diversity remains in this strain (Redman et al. 2008b; Sargison et al. 2017), it is probable that population bottlenecks, increased inbreeding, or selection have resulted in discrete regions of the genome becoming genetically fixed. Secondly, the integration of the genetic map and contiguous physical genome map allowed us to describe the recombination landscape of the genome. Although there are similarities in the recombination rate domain structure with that of *C. elegans* (Barnes et al. 1995; Rockman and Kruglyak 2009), chromosomes III and V have distinct recombination rate differences compared both to chromosomes I, II, and IV of *H. contortus*, and to all chromosomes of *C. elegans*. The broad-scale distribution is unlikely to be the result of differential recombination around centromeric sequences, given the similarities in recombination domain structure with *C. elegans* chromosomes, and that *C. elegans* chromosomes are holocentric during mitosis (Albertson and Thomson 1993; Wicky and Rose 1996). However, it has been proposed that the low or absent recombination in the chromosome termini may correlate with the presence of a spindle attachment site that guides segregation of homologous chromosomes in meiosis (Cutter et al. 2009). Although we have no data to directly test whether *H. contortus* is holocentric, we have identified low recombining chromosome termini consistent with that observed in *C. elegans*.

Despite the relatively high marker density used here (*n* = 1,618), many SNPs were completely linked in seemingly nonrecombining regions. Inclusion of a larger number of progeny would provide additional resolution to more precisely characterize variation in and transitions between recombination rate domains in each chromosome. Finally, although we could not generate a genetic map for the X chromosome due to the limited brood size and the absence of genetic diversity in the female parent, WGS data allowed us to examine genetic diversity among the female progeny, which highlighted both significant genetic variation and clustering consistent with shared paternal haplotypes in the autosomes.

### Detection of Polyandry

Technical challenges associated with single male and female mating led us to perform the genetic cross using 100 immature female MHco3(ISE) and 100 male MHco18(UGA2004) surgically implanted into the abomasum of a recipient sheep. Analysis of the genetic diversity among F_1_ progeny of a single female revealed discrete groups of progeny; given that *H. contortus* has been previously described to be polyandrous (Redman et al. 2008a), we hypothesized that these groups represented the progeny of different male nematodes. In this cross, our data support at least eight paternal genotypes contributing to multiple individuals in the brood (*n* = 41). These data are consistent with the original report of polyandry in *H. contortus*, which described at least three to four paternal microsatellite-derived genotypes from the 11 to 17 progeny sampled per single fecund female analyzed (Redman et al. 2008a). Single worm genotyping of males recovered from the initial genetic cross recipient lamb would provide further insight into the ancestral relationships among the progeny. The remarkably high frequency of polyandrous pairings would substantially increase the diversity of genotypes found among the progeny, as more possible pairs of haplotypes would be generated. This feature of *H. contortus* biology is likely to play a significant role in generating and maintaining the high levels of genetic diversity characterized in laboratory (Redman et al. 2008b) and field (Silvestre et al. 2009; Redman et al. 2015) isolates of this parasite and is also relevant to other parasitic nematode species where polyandry has been reported (Grillo 2005; Zhou et al. 2011; Hildebrandt et al. 2012).

### Detection of Nondiploid Patterns of Variation

*Haemonchus contortus* is a dioecious, sexually reproducing diploid animal. Unexpectedly, we observed 7 of the 41 progeny (17.1%) with an excess of heterozygous genotypes, and with an allele frequency spectrum that is consistent with a polyploid complement of chromosomes. Moreover, two distinct patterns of allele frequency spectrum among six of the seven putative polyploids lead us to hypothesize that these progeny arose by either: 1) nondisjunction during meiosis 1 of gametogenesis in the female parent; or 2) polyspermy, that is, an egg that has been fertilized by more than one sperm, as a consequence of polyandry (see supplementary fig. S6 and table S5, Supplementary Material online, for alternate hypotheses and evidence for the generation of triploid progeny in the brood). A third hypothesis—nondisjunction during male gametogenesis resulting in diploid sperm—was excluded; analysis of genotype frequencies among the F_1_ progeny at SNPs at which the female parent was homozygous demonstrated that paternally derived alleles from putatively heterozygous sites were segregating independently, resulting in an approximate 1:1 genotype ratio among all but one individual (supplementary fig. S2*C*, Supplementary Material online; the putative aneuploid F1_30). This supports the observation that polyploidy was inherited from diploid gametes derived from the female parent (i.e., nondisjunction), or multiple haploid gametes from the male parents (i.e., polyspermy).

Polyploidy has been previously described among nematodes. In *C. elegans*, a range of ploidy states have been characterized (see Hodgkin 2005 for review of work on natural and induced tetraploids, triploids, and haploids) and is a feature of a cellular organismal growth into late adulthood due to nuclei endoreduplication (Hedgecock and White 1985; Flemming et al. 2000). However, polyploidy is typically associated with parthenogenesis in worms (e.g., some *Meloidogyne* spp.; Lunt et al. 2014; Blanc-Mathieu et al. 2017; and some *Panagrolaimus* spp. Schiffer et al. 2017). Polyspermy in worms is thought to be rare, with a single description in the rodent filarial worm *Acanthocheilonema viteae* (McLaren 1973); more is understood in regard to the mechanisms by which polyspermy is prevented (Jaffe 1976; Wong and Wessel 2005; Johnston et al. 2010). However, polyspermy may be associated with polyandrous mating (Arnqvist and Rowe 2005), whereby sexual conflict among males (at least eight in the data presented) competing to reproduce with a female likely results in strong selection on male reproductive traits (e.g., sperm count, size, and quality), which increases the likelihood of reproductive success (Birkhead and Pizzari 2002). Although this would drive coevolution of female traits to block polyspermy, it may be that polyspermy is a consequence of this competition in polyandrous species such as *H. contortus*. Given that these progeny were sampled at the L_3_ stage, we cannot be sure that these individuals would have developed to adulthood and become reproductively viable. However, a report describing the karyotype of a single triploid *H. contortus* adult female suggests that they may be at least developmentally viable (Bremner 1954). The presence of sporadic polyploidy among the *H. contortus* F_1_ progeny represents a novel finding among parasitic nematodes; further work is required to determine if triploidy is a feature of *H. contortus* biology and prevalent in the field, or, is a novel feature of this genetic cross. If the former is true, then it will be important to be aware of ploidy variation in population genetic studies of *H. contortus*, particularly if larval stages are sampled.

A single individual (F1_30) presented with a variant allele frequency spectrum consistent with an aneuploid complement of chromosomes. Aneuploidy and other severe chromosomal abnormalities have been described in experimental hybrid crosses between *H. contortus* and the related cattle parasite, *Haemonchus placei* (Le Jambre and Royal 1980); such hybrids have recently been genetically characterized in the field (Chaudhry et al. 2015). Although such chromosomal abnormalities have not been described in within-species *H. contortus* crosses to date, the use of whole genome sequencing provides greater resolution over single marker techniques to detect these chromosome-wide changes, which may have resulted via incompatibility of rare alleles between the genetically diverse strains used in the cross.

## Conclusions

In summary, we have undertaken a comprehensive analysis of genetic diversity within a *H. contortus* family derived from an experimental genetic cross. Whole-genome sequencing of a female and her brood allowed the construction of a F_1_ genetic map, despite the challenging design dictated by the unusual biology and life history of this parasitic helminth. Development of the genetic map continues to build upon the genetic resources available for *H. contortus* as an experimentally tractable parasitic species, and provides new insight into the recombination architecture of the genome. Moreover, the close relationship between *H. contortus* and *C. elegans* argues for further comparative studies between these species. These data, together with evidence of polyandry and polyploidy, highlight the complexities of the underlying biology of *H. contortus*, and have important implications toward understanding the evolution of anthelmintic resistance in this important pathogen of livestock. Clear recombination rate differences throughout the genome will influence the rate by which a locus correlated (i.e., a genetic marker linked to resistance), or causally associated (i.e., resistance conferring mutation) with anthelmintic resistance will evolve within a population, dependent on the position in the genome that the given locus lies. Incorporating recombination rate parameters in studies that aim to genetically detect or track the transmission of resistance will be critical to the utility and interpretation of data derived from such approaches. This will be particularly the case given the likely multigenic nature of resistance to some, and perhaps all, anthelmintics. 

## Supplementary Material

Supplementary data are available at *Genome Biology and Evolution* online.

## Supplementary Material

Supplementary Figures and Tables
