# Supplementary material for: A Genome Resequencing-Based Genetic Map Reveals the Recombination Landscape of an Outbred Parasitic Nematode in the Presence of Polyploidy and Polyandry
Source: Genome Biol Evol. 2017 Dec 18;10(2):396–409. doi: 10.1093/gbe/evx269 (PMC5793844; doi:10.1093/gbe/evx269)
Supplement: Supplementary Figures and Tables [file evx269_supp.zip › Doyle_GBE_Figure_S6.pdf]

Figure S6

Hypothesis 1: Normal gametogenesis

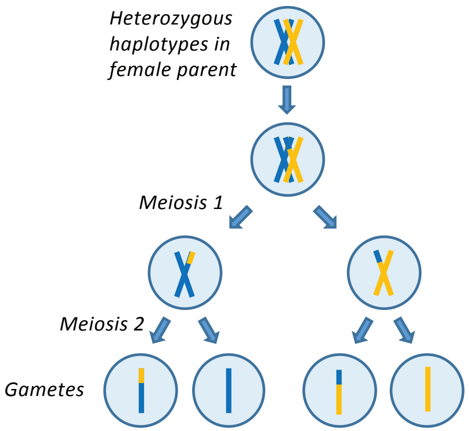

Expected and observed outcomes

1. Normal gametes produced
2. Will result in diploid progeny if fertilised with haploid sperm
3. Diploid progeny will receive a mix of parental (unrecombined) and recombinant haplotypes from parent
4. F1 progeny in cross: 34/41 progeny show this pattern

Hypothesis 2: Nondisjunction in meiosis 1

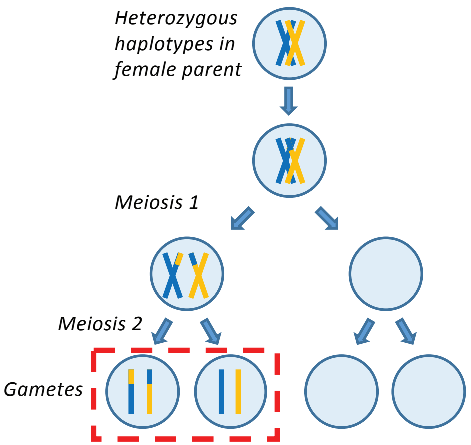

Expected and observed outcomes

1. Abnormal gametes produced -
   1/2 gametes will be diploid,
   1/2 gametes will contain no DNA
2. fertilisation of diploid gametes with haploid sperm will result in triploid progeny
3. triploid progeny will appear to be extemely heterozygous (red box) at postions in genome in which female parent was heterozygous due to inheritance of both alleles
4. F1 progeny in cross: 4/41 progeny presented with extreme heterozygosity. This scenario - nondisjunction in meiosis 1 - best explains the genetics of these triploids.

Hypothesis 3: Nondisjunction in meiosis 2

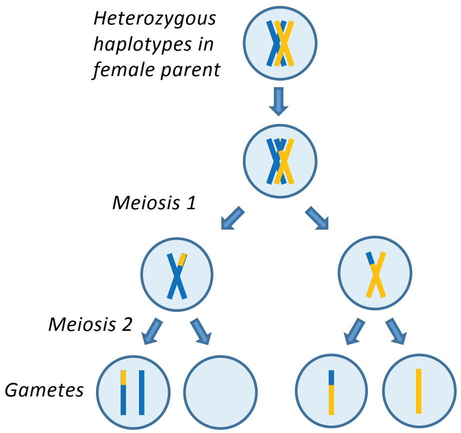

Expected and observed outcomes

1. Abnormal & normal gametes produced -
   1/4 gametes will be diploid,
   1/2 gametes will be normal,
   1/4 gametes will contain no DNA
2. fertilisation of with haploid sperm will result in triploid and diploid progeny
3. triploid progeny will appear to be mostly homozygous throughout the genome
4. F1 progeny in cross: unlikely to explain triploids in cross as there were no individuals with extreme homozygosity at SNPs in which the female parent was heterozygous

Hypothesis 4: Polyspermy

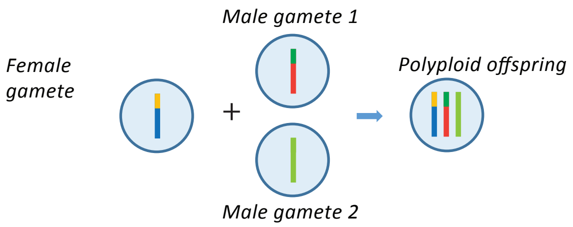

Expected and observed outcomes

1. Normal male and female gametes
2. Multiple male sperm fertilise female gamete resulting in polyploid offspring
3. F1 progeny in the cross: 2/41 progeny show triploid allele frequency pattern consistent with polyspermy
