# Supplementary material for: A Genome Resequencing-Based Genetic Map Reveals the Recombination Landscape of an Outbred Parasitic Nematode in the Presence of Polyploidy and Polyandry
Source: Genome Biol Evol. 2017 Dec 18;10(2):396–409. doi: 10.1093/gbe/evx269 (PMC5793844; doi:10.1093/gbe/evx269)
Supplement: Supplementary Figures and Tables [file evx269_supp.zip › Doyle_GBE_Table_S5.pdf]

S5 Table: Expected[1] and observed genetic consequences of triploidy via non-disjunction or polyspermy in the cross

Alternate hypotheses for observed segregation of genetic variation among F_1 progeny	Female genotype	Female gamete	Progeny genotype	Male gamete	Male genotype	Observed variant freq.[2]
**1. Pseudo-testcross (approach used for making the genetic map)**<br>• Heterozygous female, haploid gametes<br>• Homozygous male, haploid gametes<br><br>• Progeny: diploid progeny with 1:1 ratio heterozygous:homozygous genotypes<br>• Observed: 34/41 progeny show this pattern of segregation	Aa	A	AA	A	AA	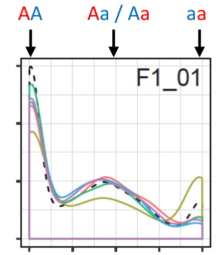
			Aa			
		a	Aa	a	aa	
			aa			
**2. Triploidy via nondisjunction**<br>• Heterozygous female with diploid heterozygous gamete<br>• Homozygous male, haploid gamete<br><br>• Progeny: triploid progeny that will appear extremely heterozygous, no homozygotes should be observed<br>• Observed: F1_21, F1_23, F1_32, F1_38 progeny show this pattern of segregation	Aa	Aa	AAa	A	AA	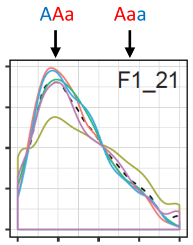
			Aaa	a	aa	
**3. Triploidy via polyspermy**<br>• Heterozygous female, haploid gametes<br>• Homozygous males, haploid gamete<br>• Multiple males (denoted by subscript 1 and 2 on male alleles) fertilise female gamete<br><br>• Progeny: multiple combinations could be produced, however, will appear as a 1:1 ratio of heterozygous (AAa/Aaa):homozygous (AAA/aaa) genotypes.<br>• Observed: F1_12, F1_40 progeny show this pattern of segregation	Aa	A	AA_1A_2	A_1	A_1A_1	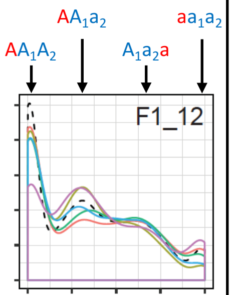
			AA_1a_2	A_2	A_2A_2	
		a	A_1a_2a	a_1	A_1a_1	
			aa_1a_2	a_2	a_2a_2	

1. Expected genotypes are described for the parents, their gametes, and the combination of parental gametes in the resulting progeny. The data is focused on heterozygous female and homozygous male variants, as this was part of the criteria for selecting variants to be used in the genetic map, i.e., SNPs must be heterozygous in the female and segregate in a 1:1 genotype ratio in the progeny
2. A single example of the observed genetic variation associated with each hypothesis is presented. Variant frequency data for all progeny is presented in Figure S3.
