# Supplementary figures and images for: A Genome Resequencing-Based Genetic Map Reveals the Recombination Landscape of an Outbred Parasitic Nematode in the Presence of Polyploidy and Polyandry
Source: Genome Biol Evol. 2017 Dec 18;10(2):396–409. doi: 10.1093/gbe/evx269 (PMC5793844; doi:10.1093/gbe/evx269)

Figure S1

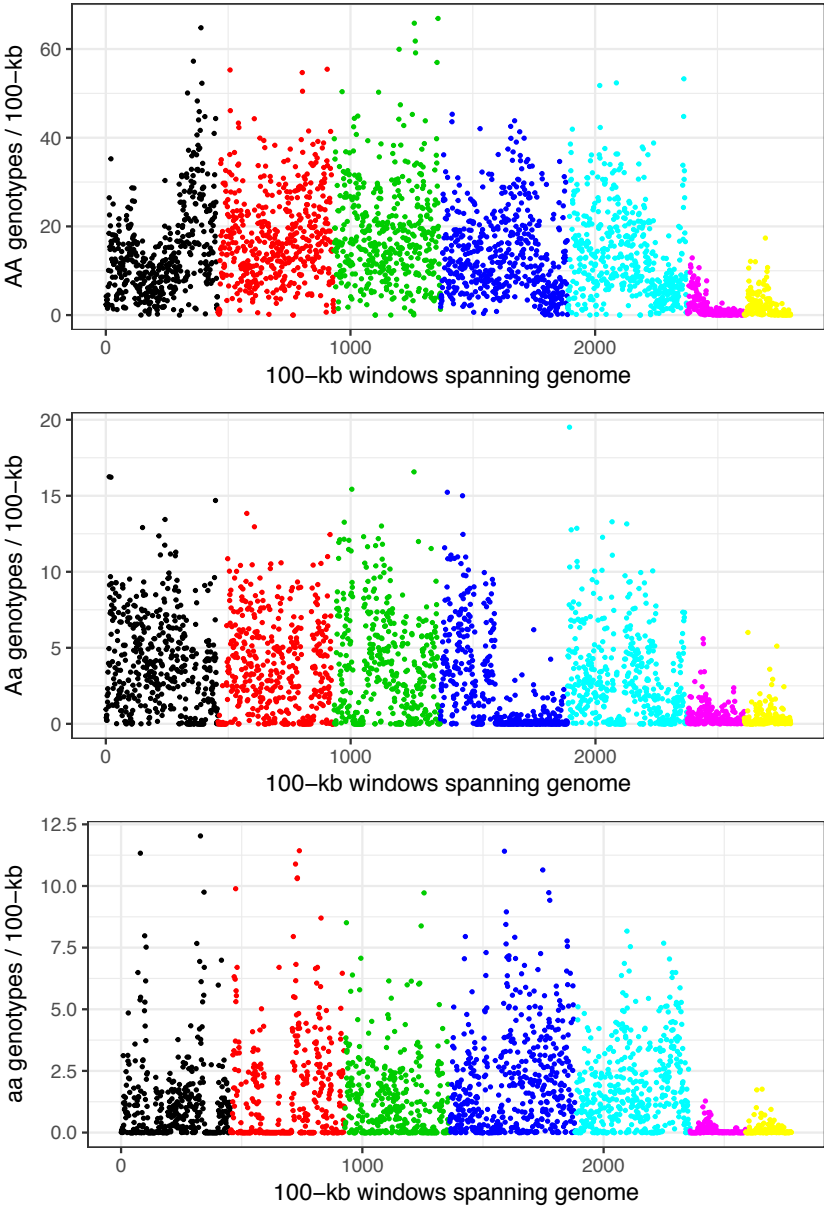

Supplement: Supplementary Figures and Tables [file evx269_supp.zip › Doyle_GBE_Figure_S1.pdf]

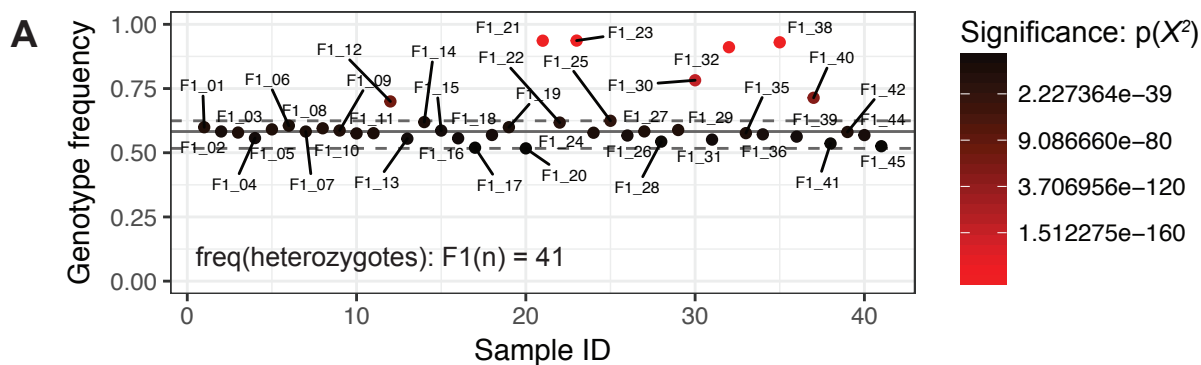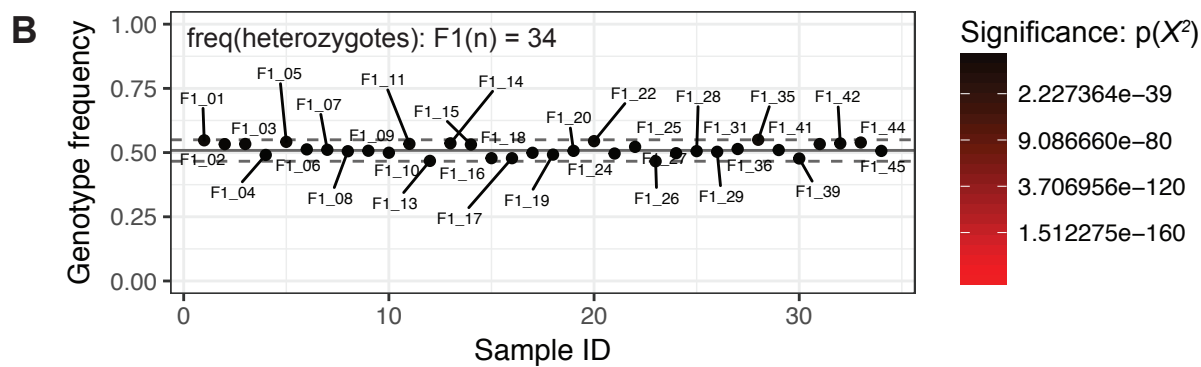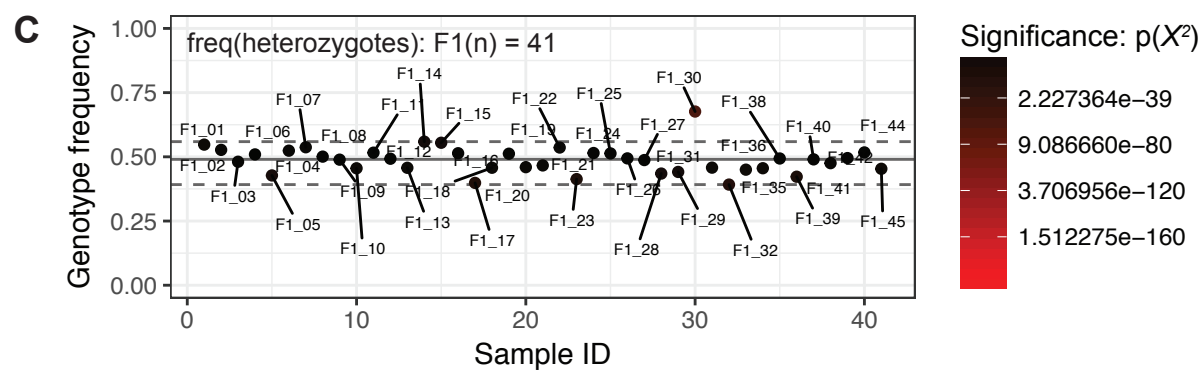

**C**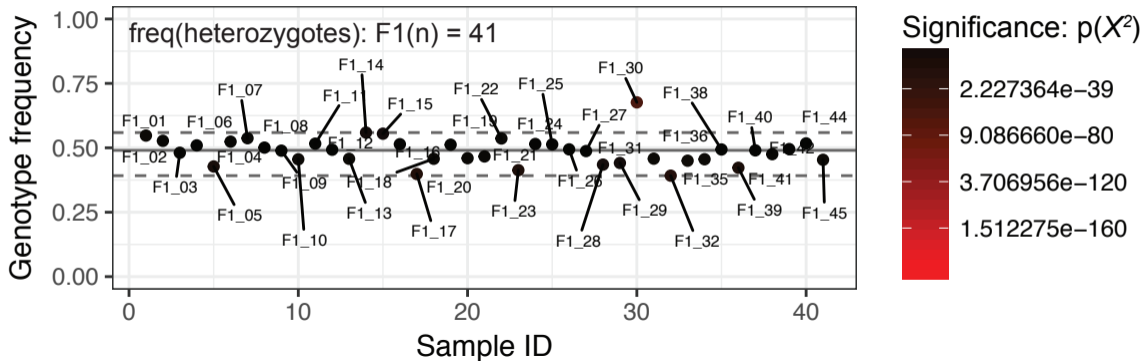

Supplement: Supplementary Figures and Tables [file evx269_supp.zip › Doyle_GBE_Figure_S2.pdf]

Figure S3

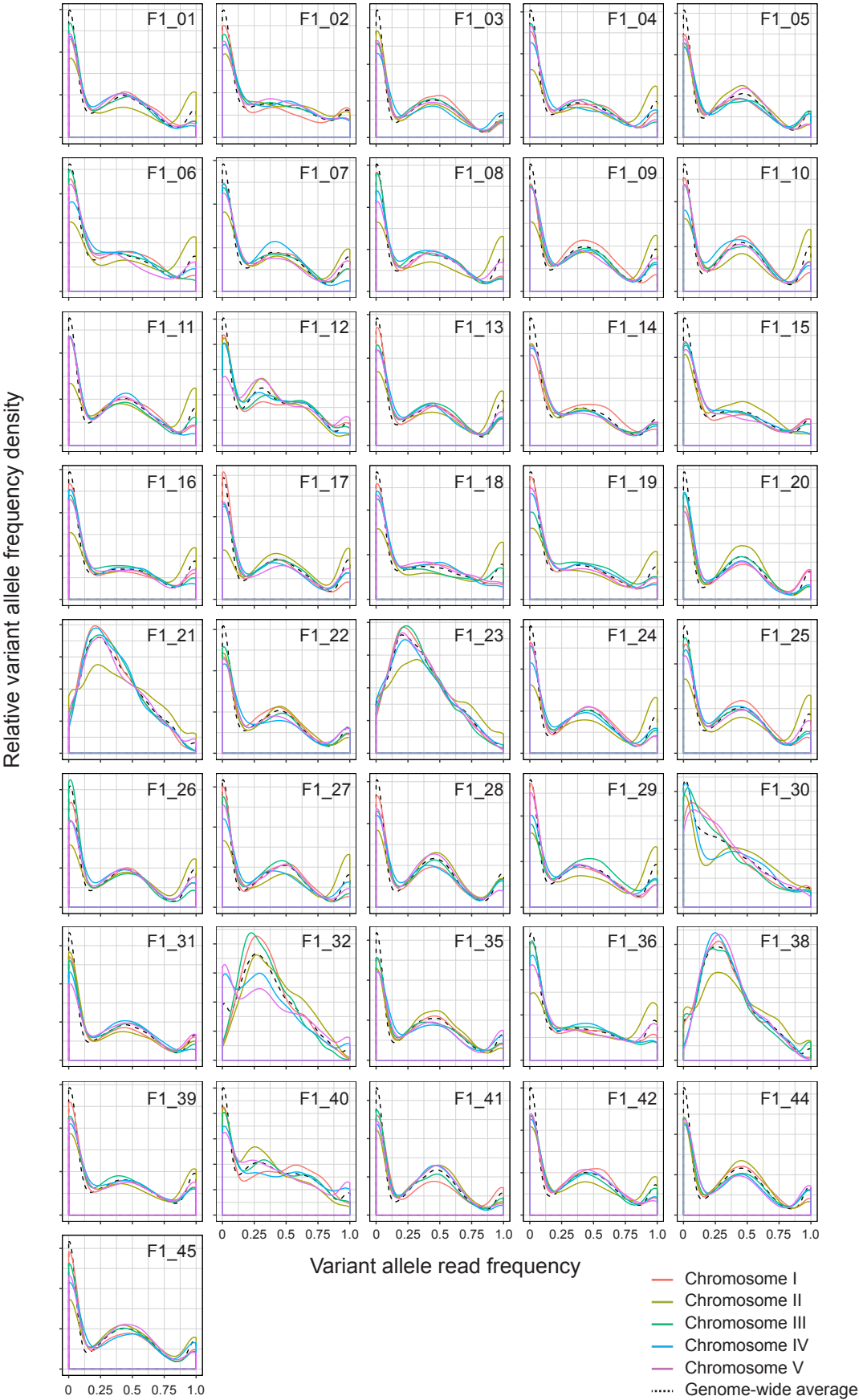

Supplement: Supplementary Figures and Tables [file evx269_supp.zip › Doyle_GBE_Figure_S3.pdf]

Figure S4

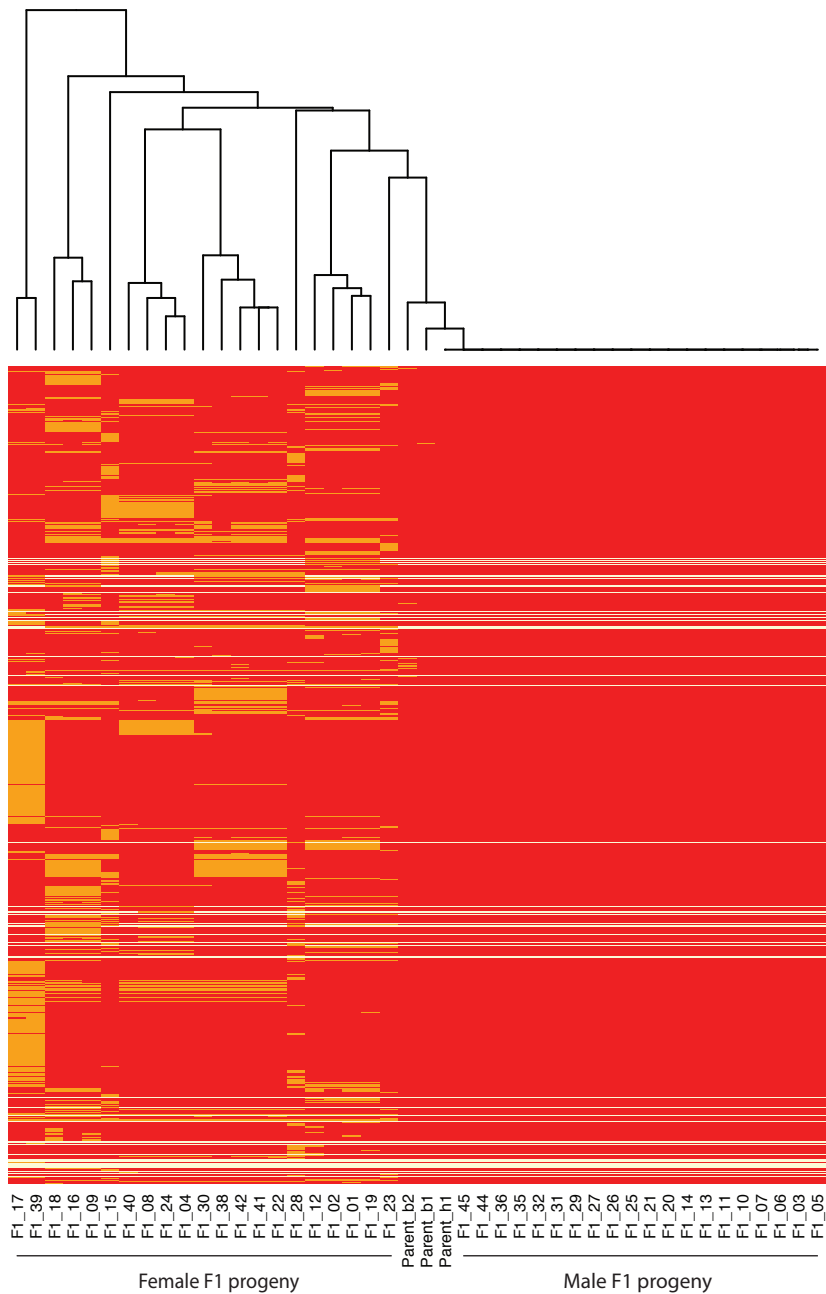

Supplement: Supplementary Figures and Tables [file evx269_supp.zip › Doyle_GBE_Figure_S4.pdf]

Figure S5

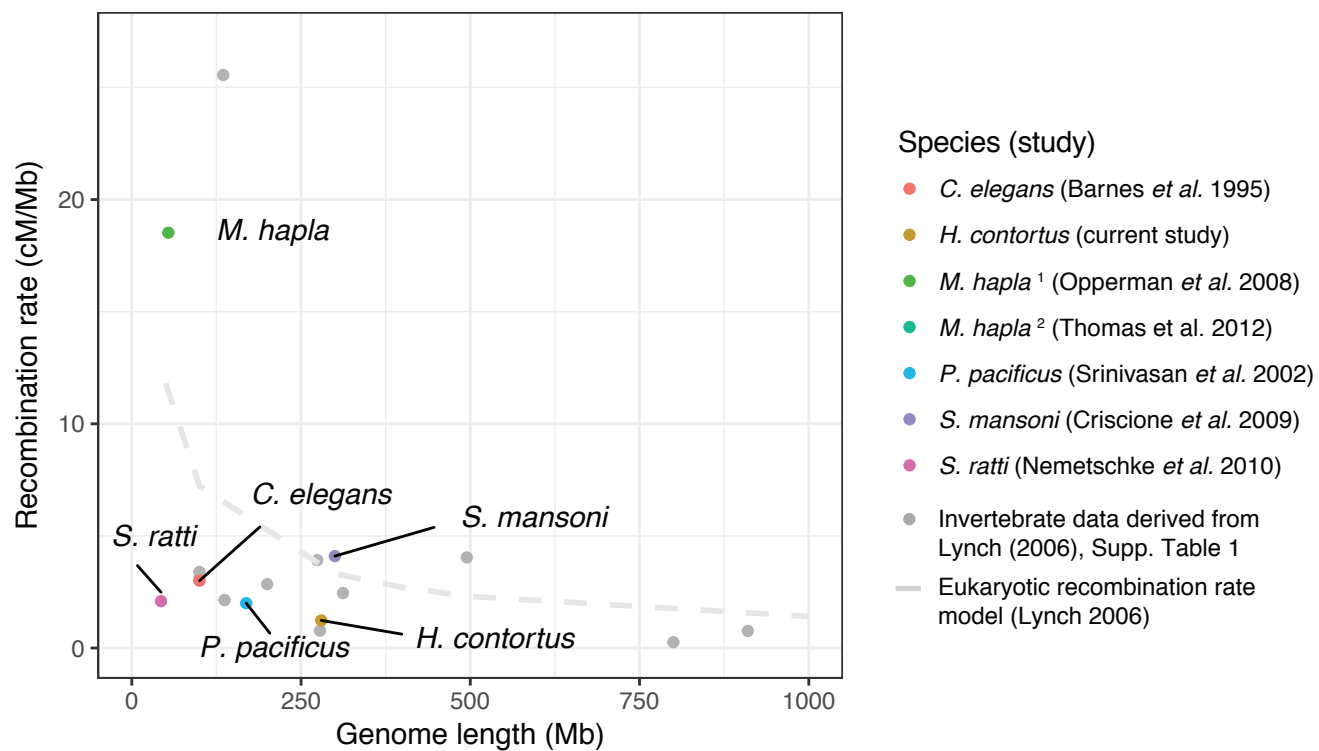

Supplement: Supplementary Figures and Tables [file evx269_supp.zip › Doyle_GBE_Figure_S5.pdf]
